# Notes on Preparations for the Sick

**Published:** 1887-06

**Authors:** 


### Notes on Preparations for the Sick.

---

**Pharmaceutical Preparations.** Burroughs, Wellcome and Co., London.

We have received from this firm a large number of their special preparations, of which we are able to speak in the highest terms.

Amongst the dietetic group we have to notice the following:

**Zymine (Fairchild), Extractum Pancreatis.**
**Peptonising Powders (Fairchild).**
**Zymine Tabloids ,,**
**Compound Zymine Tabloids (Fairchild).**
**Pepsine in Scales ,,**
**Pepsine Tabloids ,,**

In the preparation of foods for the sick these excellent digestives are now taking a prominent position. The time is doubtless far distant when, as suggested by some facetious writer, the organism, "lightened of its load of viscera," may "go on its way rejoicing" by the aid of special chemically-prepared foods needing no digestion; nevertheless, the predigestion of food is far too important a matter to the sick to be passed over lightly by the physician, and the effects of predigestion are so marked that the patient who needs artificial aid will not be deterred by the scoff of the sceptic from adopting a method which is easily tested and is not usually found wanting.

Although we hold that the special utility of the peptonising process arises from its applicability to the *preparation of milk* for those subjects who cannot digest casein, or where the presence of curdled casein in the stomach might be deleterious; yet other peptonised foods, such as milk gruel; ox tail, mock turtle, and other soups; beef-tea, jellies, custards, blanc-manges, puddings, &c., are easily prepared with the pancreatic powder, and are found to agree in many aggravated cases of stomach disease when every other kind of treatment fails.

In any case of chronic dyspepsia it will suffice to supply only peptonised milk, and the patient will usually improve: most of the symptoms of acute and chronic dyspepsia are relieved; eructations, nausea, vomiting, pain, and flatulence usually cease, and ere long the patient is able to take first some other variety of peptonised and then unpeptonised food. In cases of acute febrile disease, such as enteric fever, if there should be vomiting, or any tendency to meteorism, the predigestion becomes a necessity which rarely fails to give as good a result as could fairly be expected from it.

We have found the **Extractum Pancreatis** (Fairchild) to be a most efficient peptonising agent. It is a dry powder, containing all the pancreatic enzymes in a most active and concentrated form, put up in convenient glass tubes as peptonising powders, with instructions which even the inexperienced can scarcely fail to understand: it is therefore an efficient agent for the preparation of predigested foods.

The **Tabloids of Zymine** are said to be the most convenient form for internal administration, for the purpose of aiding intestinal digestion. They should be swallowed either immediately after or two hours after a meal: in the former case they are considered to aid acid digestion in the stomach; in the latter case they may pass safely through the acid contents of the stomach during the later phases of digestion, when the extreme acidity diminishes, and reach the alkaline area beyond, where they will aid the pancreatic fluid in its various functions. Certainly we have found that the internal administration of pancreatine in powder or tabloids does relieve the symptoms of intestinal dyspepsia, more especially the flatulence of the torpid alimentary canal of senility. Each tabloid contains three grains of zymine in a form agreeable to the taste, easily carried about in the pocket, and easily swallowed.

The **Compound Zymine Tabloids,** containing three grains of bismuth subnitrate and a small dose of powdered ipecacuanha, with two grains of zymine, form a convenient and useful combination in many cases of weak digestion.

The **Pepsine Tabloids** and the **Pepsine in Scales** are both efficient substances in aiding gastric digestion. One grain of the latter will digest one thousand grains of egg-albumen, and one to three grains is a sufficient dose. Each tabloid contains one grain of the pepsine in scales, together with acids and appropriate aromatics: they can easily be swallowed entire, but are not unpalatable if broken or sucked away. The advantages of this method of administration over the nauseous powders, or the liquid pepsines, are sufficiently obvious.

Another group of partly dietetic, and partly medicinal, substances, is the following:

**Kepler's Extract of Malt.**
**" " " with Cod Liver Oil.**
**Beef and Iron Wine (Burroughs).**
**" " " " with Quinine (Burroughs).**
**Kepler's Cod Liver Oil.**

The composition and utility of **Malt Extracts** are now well understood. Prepared at a temperature which never reaches 90°, the diastatic ferment has not been destroyed, and remains in combination with the malt-sugar, phosphates, dextrines, and albuminoids of the malted barley. The combination is therefore a valuable food, and at the same time an aid to digestion: it is pre-eminently a brain and nerve food, containing tissue-forming as well as force-producing and warmth-giving elements, in a form which will not tax digestion and will be easily assimilated.

The **Malt Extract with Cod Liver Oil** is spoken of as a solution, rather than a mixture or emulsion: "the oil is completely dissolved, so that globules, even as small as those in milk, cannot be seen." We have examined this "solution" under the microscope: no oil globules are visible, but we altogether decline to consider this mixture of two substances with the same refractive index as a solution. The mixture is homogeneous, and perfectly transparent even with high powers, the one being absolutely invisible in the presence of the other; but this is clearly due to the absence of any difference of refractive power of the two substances: we cannot admit that the oil has been dissolved in the watery extract. An apparent solution has been made; but in reality the combination must be looked upon as an emulsion, and hence it has the advantages arising from minute subdivision of the oil. It is well known that plain oil is apt to derange the digestive functions, to cause nausea, and sometimes diarrhœa, the oil passing through the alimentary canal undigested and unabsorbed. It has been said that greater benefit will usually be derived from five pounds of malt and oil than from twenty-five pounds of oil alone. Certainly the combination is more easily digested, and forms a highly nutritious, force-giving and heat-giving food. Its great utility in wasting diseases of all kinds, more particularly in scrofulous derangements, is now generally recognised; and its superiority over the ordinary emulsions of cod liver oil is apparent, not only as regards its nutritive elements, but as a diastatic aid to digestion, and its agreeable taste as compared with other oily mixtures.

The **Burroughs Beef and Iron Wine**, with or without Quinine, is a combination of considerable nutrient and at the same time medicinal value. We have given them frequently in cases of convalescence from acute disease, and with decided benefit. Where active medication is not necessary, and where, as in the case of children, the ordinary stimulants may well be avoided, these restorative fluids expedite convalescence, and take the place of other more nauseous medicines. We have given them of late in many cases of measles, after the temperature had become normal, and with very good results. Their taste is not suggestive of chemical manipulation—even children take them readily; we think them excellent forms of medicinal food.

One of the most important groups of medicinal substances prepared by this firm is the **Hypodermic Tabloids.** These are well known to be accurate, efficient, and convenient. For some years we have given up anything else for hypodermic medication: the absolute freedom from change and constancy in effects have given them a position superior to all the various solutions which have been in use. The morphia tabloids never fail to act with rapidity, and commonly without subsequent vomiting: formerly we found that with a glycerine solution vomiting was the rule, now it is quite the exception. On two occasions we have observed rather alarming effects from these tabloids—in the one case with aconitin, in the other with pilocarpin. The first case was an urgent one. Morphia had failed to arrest a chronic sciatica, and it was thought that a full dose of aconitin hypodermically would be likely to relieve. The patient was an adult man, accustomed to active medication: accordingly two of the stronger tabloids were dissolved, and the whole quantity (gr. $\frac{1}{65}$) injected into the painful nerve. In the course of twenty minutes the patient complained of the earlier symptoms of aconitin, tingling in tongue and in fingers, then the pulse became intermittent, afterwards the pulse failed entirely, the patient becoming cyanotic and unconscious: for four hours he was in imminent peril, and then gradually recovered.

The other case was a severe attack of catarrhal asthma. Two of the $\frac{1}{10}$th grain tablets of pilocarpin were injected hypodermically, after several Joy's cigarettes had been smoked, and several doses of chloral and stramonium given. Two hours later the pulse was scarcely perceptible, the patient was unconscious, was perspiring freely, and had a little salivation. These symptoms passed off a few hours later, and the patient convalesced rapidly, but for several hours had been in a critical condition, requiring hypodermic injections of ether.

These cases, and some others, suggest the reflection that for hypodermic injection excessive caution in dose is needful; should there be an idiosyncrasy on the part of the patient, the drug once injected cannot be removed, but can only be counteracted by some antidote.

The complete hypodermic pocket-cases with a selected series of tabloids form excellent companions when called to an emergency in the night: everything needful for the immediate relief of most conditions of emergency will be always ready.

The following newer tablets have been forwarded, and will doubtless be of occasional utility:

Quinine Hydrobromate, gr. $\frac{1}{2}$.

Aloin, gr. $\frac{1}{4}$.

Homatropin, gr. $\frac{1}{250}$.

Codein Phosphate, gr. $\frac{1}{4}$.

Caffeine Sodio-salicylate, gr. $\frac{1}{2}$.

Colchicin, gr. $\frac{1}{100}$.

The range for the use of such tablets as these hypodermically must of necessity be limited; but for internal administration they are a convenient and portable form in the treatment of emergencies where hypodermic medication is unnecessary.

The well-known series of **Wyeth's Compressed Tablets** has recently received several important additions, of which the most important are the following:

Antifebrine, grs. 2.
Antipyrine, grs. 5.
Bismuth Subnitrate, grs. 5 and 10.
Manganese Binoxide, grs. 2.
Dover's Powder, grs. 5.
Pulv. Ipecacuanha, grs. 5.
Urethane, grs. 5.
Strophanthus = two minims.
Trinitrine, gr. $\frac{1}{50}$ and $\frac{1}{100}$.
Comp. Trinitrine (containing gr. $\frac{1}{4}$ of Amyl Nitrite), gr. $\frac{1}{100}$.
Voice Tabloids (Cocaine, with Potass. Chlorat. and Borax).

These contain the pure drug only, without admixture or excipient, and are a convenient and portable form in which to keep a few important drugs in measured doses ready for cases of emergency. A table of poisons and their antidotes has been drawn up, and an antidote case containing all the necessary tablets and tabloids is prepared by this firm.

We have found the Urethane tablets to be perhaps the most frequently useful of the whole series. One, two, or three may be given as a hypnotic at bedtime, and others at intervals if they should be required. One patient has taken as many as ten during one night; but it rarely happens that more than three or four are necessary to secure a good night's rest.

---

**Fluid Extract of Cascara Sagrada. Cascara Cordial.** PARKE, DAVIS, & CO., Detroit, U.S.A.

The discovery of the specific action of the Cascara Sagrada on the muscular coat of the intestine, and its power of imparting tone and elasticity to the relaxed intestinal walls, thus restoring the natural vermicular movement and radically relieving habitual constipation, has elevated this new drug to a position which few have attained in so short a time. It appears to be generally admitted that this preparation is now the most important remedy in the treatment of habitual constipation: it is an aperient, but this is not all; it is *the* aperient which appears to have almost taken the place of all others where only a gentle stimulant to peristaltic action is required.

The most eligible form for the administration of the drug is that offered under the name of Cascara Cordial, in which the medicinal action of the drug is fully preserved, while its inherent bitter qualities have been so skilfully disguised by carminatives and aromatics as to render it acceptable even to children and those who commonly refuse unpalatable medicines. The dose as a laxative is from one-fourth to one teaspoonful, increased if necessary, twice a day, night and morning. The minimum dose should be commenced with, and gradually increased; and then, after continuing several months, should be discontinued in gradually decreasing doses. The object of treatment should be rather to permanently remove the cause than to temporarily relieve the symptom.

We have now and then found a patient who required doses of half-an-ounce or more: in such cases the addition of a few drops of the fluid extract will generally suffice to produce the desired result, or the fluid extract alone may be given in place of the cordial. Various combinations, such as cascara and euonymin, or cascâra with rhubarb and nitro-muriatic acid, have been found to be efficient; and the cordial forms a useful and efficient vehicle for quinine and other remedies.

We have reason to think that the preparations of Messrs. Parke, Davis, and Co. are distinctly superior to others which are frequently dispensed and sometimes fail.

---

### Mineral and Medicinal Non-alcoholic Beverages.

CARTER & CO., Bristol.

**Carter's Bristol Soda Water** has long been known as one of the most highly charged with carbonic acid. The purity of the Bristol water has recently been submitted to the most severe and hostile tests; but no serious evidence could be obtained as to the presence of any deleterious organic impurity: it is, however, of interest to know that careful experiments show that if through inadvertence, ignorance, or carelessness any micro-organisms should remain in the water to be bottled, they are quite destroyed, and the water sterilised, in proportion to the volume of carbonic acid and the longer such microbes are brought under its influence. The water supplied by the Bristol Company, brought from the Mendip Hills, has been shown to be one of the best of its kind for drinking purposes; and when highly charged with carbonic acid by means of the most perfect machinery, with every guarantee of freedom from all injurious metallic salts, becomes one of the most perfect table waters which either nature or art can procure. We are often told by patients that soda water is "lowering"; but we have had no evidence of any such result, and believe it to be in opposition to universal experience. We think that this beverage is one of the most wholesome, refreshing, and exhilarating means of washing away the products of physiological tissue-activity, and that its abundant use at home would frequently take away the need for lavish expenditure of time and money in visiting the various foreign health resorts, in which the drinking of an aerated water forms the most essential element of treatment.

The **Lime Fruit Flavoured Soda Water** is a mixture of the ordinary soda water with lime fruit syrup, and is a very palatable combination for those who like this kind of drink.

The **Orange Champagne** is a non-alcoholic fluid made from Seville oranges; and the

**Ginger Champagne** from fine Jamaica ginger. These fluids are elegantly mounted in champagne bottles, and are both agreeable substitutes for the alcoholic fluids which formerly occupied those bottles. They cannot fail to be appreciated by abstainers who prefer the stimulating action of ginger, and by dipsomaniacs who can drink nothing from any other kind of bottle.

The **Tonic Quinine Water** contains Howard's sulphate of quinine with a little fruit liqueur. We prefer to take our medicine in the form of coated pills.

---

**St. James' Rum.** G. W. CHRISTIE & Co., London.

We have received a sample of this very pleasant and pure spirit, which a medical man will find exceedingly useful when he wishes to administer such a stimulant.

